# Vaccine Acceptance in Patients with Inflammatory Bowel Disease: Lessons Learned from the COVID-19 Pandemic

**DOI:** 10.3390/vaccines12050551

**Published:** 2024-05-18

**Authors:** Giada Mastrangeli, Filippo Vernia, Stefano Necozione, Mario Muselli, Sara Frassino, Nicola Cesaro, Giovanni Latella, Leila Fabiani

**Affiliations:** 1 Department of Life, Health and Environmental Sciences, University of L’Aquila, 67100 L’Aquila, Italy; giada.mastrangeli@graduate.univaq.it (G.M.); stefano.necozione@univaq.it (S.N.); mario.muselli@univaq.it (M.M.); leila.fabiani@univaq.it (L.F.); 2 Division of Gastroenterology, Hepatology and Nutrition, Department of Life, Health, and Environmental Sciences, University of L’Aquila, Piazzale S. Tommasi, 1-Coppito, 67100 L’Aquila, Italy; filippo.vernia1@gmail.com (F.V.); sara.frassino@graduate.univaq.it (S.F.); dott.nicolacesaro@gmail.com (N.C.)

**Keywords:** vaccine acceptance, SARS-CoV-2, COVID-19, vaccination, inflammatory bowel disease (IBD)

## Abstract

Background: Immunomodulating therapies, which are commonly used in patients with Crohn’s disease (CD) and ulcerative colitis (UC), have been linked to an increased risk of contracting opportunistic infectious diseases, the majority of which are preventable through vaccination. Nonetheless, vaccination rates in these patients are suboptimal, and frequently lower than in the general population. The COVID-19 immunization schedule provided a new scenario for investigating vaccine acceptance in patients with inflammatory bowel disease (IBD), with uncertainty and concerns emerging and the number of subjects receiving the third and fourth doses of the vaccine gradually decreasing. This study investigated IBD patients’ attitudes towards previous COVID-19 vaccine programs and identified the factors that influence their adherence. It considered demographic and disease-related factors as well as the role of gastroenterologists and primary care physicians (PCPs). Methods: Data were collected through a self-completed questionnaire administered to all adult IBD patients (age > 18) who visited the Gastroenterology, Hepatology, and Nutrition division at the University of L’Aquila (Italy) for a regular follow-up between November 2021 and December 2022. Non-IBD gastroenterological outpatients who visited during the same period were included as a control group. Results: A total of 178 patients were included in the analysis. The IBD group consisted of 77 patients, 48.1% with CD and 51.9% with UC; the mean age was 49.5 years and 51.9% were female. Overall, 94.8% of IBD patients had undergone at least one vaccine dose and 79.2% had received two doses, versus 8% of the control group (*p* < 0.0001). A total of 84.4% of IBD patients reported their propensity towards COVID-19 vaccination, with an average agreement score significantly higher than the controls (*p* = 0.0044). The trust of IBD patients in the effectiveness of the COVID-19 vaccine (*p* < 0.0001) and its role in hastening pandemic resolution (*p* < 0.0001) is strongly related to motivation and propensity. Concerns about the safety of the COVID-19 vaccine in IBD (*p* = 0.0202) and fear of vaccine-induced flare-ups (*p* = 0.0192) were reported as the main barriers. No correlation was found between COVID-19 vaccine propensity and clinical features like the type of IBD, years of disease, activity, and ongoing treatment. Regarding the recommendations received from physicians to get vaccinated against COVID-19, IBD patients relied heavily on their gastroenterologists for advice, while the control group relied mainly on their PCPs. Conclusions: The overall positive attitude towards vaccinations reported in our study was better than that observed for other vaccines. The relationship of trust with the gastroenterologist should be used to boost vaccination against other preventable diseases in IBD patients. Our findings add information on the factors influencing vaccine propensity, which can be used to improve current vaccination strategies.

## 1. Introduction

Inflammatory bowel disease (IBD), including ulcerative colitis (UC) and Crohn’s disease (CD), is a chronic relapsing condition of the gastrointestinal tract. The prevalence in Europe ranges between 50–54/100,000 people for CD and 50–80/100,000 people for UC. Both cause significant morbidity and impact patients’ quality of life [1,2].

A large proportion of inflammatory bowel disease patients need immunomodulating therapies throughout their lives, which are associated with a higher risk of contracting opportunistic infectious diseases [3,4,5]. Most of these infections, including hepatitis B (HBV), flu, pneumococcal pneumonia, herpes zoster virus (HZV), and human papilloma virus (HPV) infection, can be prevented by vaccination [6]. Thus, before treating patients with immunomodulators, guidelines suggest investigating vaccination status and completing vaccination schedules when required [6,7,8]. Nonetheless vaccination rates are suboptimal, often lower than in the general population [9,10].

The exponential spread of severe acute respiratory syndrome coronavirus 2 (SARS-CoV-2) represented a testing ground for developing a comprehensive immunization plan to achieve herd immunity and reduce COVID-19-related deaths and hospitalizations, more so in frail categories of patients.

According to available data, patients with CD and UC are not at an increased risk of acquiring or developing severe SARS-CoV-2 infection [11,12]. However, some medications used in IBD, such as systemic corticosteroids, are linked to an increased risk of worse COVID-19 outcomes, including hospitalization, intensive care unit (ICU) admission, or death [13]. Although IBD patients treated with immunosuppressant and immunomodulating therapies were excluded from Phase III clinical trials aimed at establishing vaccine safety and efficacy, the main gastroenterology societies promptly released position statements encouraging the use of COVID-19 vaccines in this population, comparing the potential benefits and drawbacks of immunization [14,15].

The safety and efficacy of COVID-19 vaccines in IBD patients has been proven, as the incidence of serious adverse events is comparable to that of other cohorts of patients [16]. However, uncertainty and concerns emerged, as undocumented news released by active anti-vaccine groups contributed to an increase in patients’ worries about the possible risk of immunization and/or negative effects on their medical condition, undermining COVID-19 vaccine uptake rates.

Vaccine acceptance from IBD patients thus became a crucial task to deal with, as the number of subjects undergoing the third and fourth dose of the COVID-19 vaccine progressively declined. The time-related reduction of vaccine effectiveness, and new virus variants constantly appearing even in the face of a less severe manifestation of the virus, will further change the clinical scenario in the next few months.

This cross-sectional study was aimed at investigating the attitudes and beliefs of IBD patients during previous COVID-19 vaccine programs and assessing the main determinants of their propensity or avoidance to adherence. The relative weight of demographic- and disease-related factors, as well as the intervention of gastroenterologists and primary care physicians (PCPs), have been taken into consideration for a better understanding of patients’ attitudes and potential influencing factors needed to implement the next targeted vaccination-encouraging strategies and increase vaccination rates.

## 2. Materials and Methods

### 2.1. Study Design and Patients

Data were collected using a self-completed questionnaire. All adult patients (age > 18) with IBD who attended the Gastroenterology, Hepatology, and Nutrition division at the University of L’Aquila (Italy) for a regular follow-up from November 2021 to December 2022 were recruited and asked to participate in the survey. Non-IBD gastroenterological outpatients who visited during the same period were included as a control group. All consecutive patients attending the division during the study’s time frame were sequentially enrolled. Patients were provided with pertinent information about the study’s general topic; medical and nursing staff were available to answer any questions about the questionnaire. All patients gave their informed consent and data were collected anonymously.

Ethical approval was obtained by the Internal Review Board of the University of L’Aquila (protocol code 131090, 22 November 2021), in accordance with the principles laid out in the Declaration of Helsinki.

### 2.2. Exclusion Criteria

Age < 18 years, previous diagnosis of cancer or psychiatric diseases (excluding depression), HIV infection, and pregnancy were considered exclusion criteria.

### 2.3. Questionnaire Items

A questionnaire based on the previous literature and used in similar studies was developed for the purpose of this study, in line with its aims [17,18,19,20]. The tool consisted of 20 questions and provided details on the patients’ demographics (sex, age at recruitment, level of education, and employment status); self-perceived health; IBD medical history (type of IBD, disease onset, disease extension and activity, and current medication); personal history of SARS-CoV-2 infection; COVID-19 vaccination status and regular immunization for seasonal influenza in the last five years; attitudes and perceptions toward vaccines in general; factors related to COVID-19 vaccine acceptance.

The final section of the questionnaire addressed the main sources of information and the clinicians’ role in recommending the vaccination.

Disease activity was evaluated using the Harvey–Bradshaw Index (HBI) in CD and the clinical Mayo Score (PMS—Partial Mayo Score) in UC. Patients were classified according to the disease status as quiescent, mild, moderate, and severe. The risk of COVID-19 was assessed with the BSG risk grid [21].

Attitudes, opinions, and beliefs were ranked based on the scoring of specific statements on a 5-point Likert scale (0 = no opinion, 1 = strongly disagree, 2 = disagree, 3 = agree, and 4 = strongly agree). The average agreement score was calculated for each item. Other statements were investigated through multiple-choice questions.

### 2.4. Statistical Analysis

The normality of the distributions of variables was evaluated with the Shapiro–Wilk test. As appropriate, Student’s *t*-test or Wilcoxon–Mann–Whitney test were used to analyze continuous variables that were reported as the mean and standard deviation (±SD). Fisher’s exact test or a chi-square test were used to determine variations in frequencies among groups for categorical variables, which were stated in terms of the number of observations or percentages.

Spearman’s rank-order correlation analysis was performed to quantify the strength and direction of association between COVID-19 vaccine propensity and the investigated determinants.

Statistical significance was set at *p* < 0.05. All the statistical analyses were performed using SAS version 9.4 (SAS Institute Inc., Cary, NC, USA).

G*Power software 3.1 was used to perform a post hoc power analysis (https://www.psychologie.hhu.de/, accessed on 20 March 2024). The study had a sufficient sample size of cases and controls to achieve 89% power to detect a medium effect size (Cohen’s d = 0.50) with a type I error of 0.05 on the vaccine propensity between the groups.

## 3. Results

### 3.1. Patients’ Sociodemographic and Clinical Characteristics

A total of 178 patients were included in the analysis. Table 1 provides the main demographic and social characteristics of the study population.

The IBD group consisted of 77 patients, 37 (48.1%) with CD and 40 (51.9%) with UC; the mean age was 49.5 years and 51.9% were female. The clinical features of IBD patients are reported in Table 2.

### 3.2. Assessment of COVID-19 Vaccine Uptake

The majority of IBD patients was vaccinated against COVID-19, with a variable number of doses according to the Italian immunization schedule.

Overall, 94.8% (73 of 77 IBD patients) had undergone at least one vaccine dose. At the time of the interview, 79.2% of IBD patients had received two doses versus 8% of the control group (*p* < 0.0001). Four IBD patients (5.2%) were not vaccinated, compared to zero in the control group (Table 3).

Only one third of the IBD cohort had received regular immunization for seasonal influenza in the last five years (33.8% vs. control group 66.2%, *p* = 0.082), despite comparable mean age.

Most patients reported that they had not contracted COVID-19 (IBD 88.3% vs. 71% control group, *p* = 0.0464) but personally knew someone who had (IBD 62.3% vs. 77.5% control group). A small proportion of patients were diagnosed with COVID-19, ranging from asymptomatic infection to hospitalization (IBD 11.7% vs. 29.0% control group). The proportion of mild cases was significantly different in the two groups (IBD 5.2% vs. 22.0% control group) (Table 3).

### 3.3. Attitudes and Beliefs towards Vaccinations in General

The majority of IBD patients (89.6%) agreed on the importance of vaccines in preventing potentially severe diseases (score 3.45 SD ±0.89 vs. 3.31 SD ±1.0 control group). However, 31.2% reported being worried about the impact of vaccination on their medical condition (score 1.87 SD ±1.21), with 29.9% also expressing concerns about the potential long-term effects (score 1.90 SD ±1.17).

Only four IBD patients (5.2%) agreed with the statement that vaccines are useless or even harmful (score 1.23 SD ±0.72 vs. 1.23 SD ±0.72 control group), but a larger proportion (27.3%) believed that acquired immunity through natural infection is more beneficial than vaccination (score 1.50 SD ±0.78 vs. 1.60 SD ±1.02 control group).

In relation to the above data, there was no significant statistical difference between IBD patients who had received vaccinations and those who had not, nor between cases and controls.

### 3.4. COVID-19 Vaccine Acceptance: Propensity and Barriers

COVID-19 vaccine propensity was defined as having answered “agree” or “strongly agree” to the statement “I am in favor of the COVID-19 vaccination and have received at least one dose or I am willing to vaccinate”.

**Figure 1:**
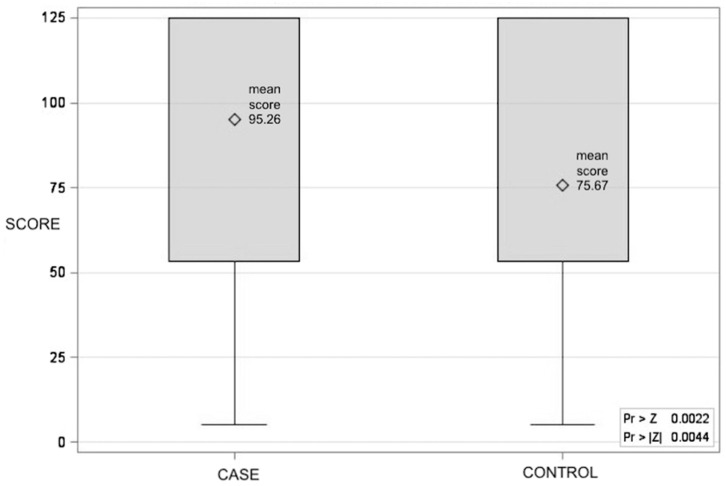
The Wilcoxon rank-sum score distribution for COVID-19 vaccine propensity between case and control groups (CASE: mean score 95.26; CONTROL: mean score 75.67; *p* = 0.0044).

Out of 77 IBD patients, 65 (84.4%) reported their propensity towards COVID-19 vaccination, with an average agreement score significantly higher than the controls (score 3.45 SD ±0.95 vs. score 3.04 SD ±1.14 control group, and Wilcoxon rank-sum scores of 95.26 vs. 75.67, *p* = 0.0044) (Figure 1).

As reported in Table 4, a further analysis investigated several determinants correlated to COVID-19 vaccine propensity.

The trust of IBD patients in the effectiveness of the COVID-19 vaccine (0.77, 95% CI 0.65–0.85, *p* < 0.0001) and its role in hastening pandemic resolution (0.73, 95% CI 0.60–0.83, *p* < 0.0001) are strongly related to motivation and propensity.

A positive correlation was observed between COVID-19 vaccine propensity and overall trust in vaccines’ effectiveness against serious illness (0.60, 95% CI 0.43–0.73, *p* < 0.0001).

Concerns about the safety of the COVID-19 vaccine in IBD (−0.28, 95% CI −0.48 to −0.04, *p* = 0.0202) and fear of vaccine-induced flare-ups (−0.28, 95% CI −0.48 to −0.04, *p* = 0.0192) were reported as the main barriers with a mild correlation.

Receiving the COVID-19 vaccine was not related to propensity in IBD patients or controls, but it might act as a barrier (0.16, 95% CI −0.06 to 0.38 vs. −0.27 95% CI −0.27 to −0.08, *p* = 0.002).

No correlation was found between COVID-19 vaccine propensity and the type of IBD, as well as clinical features like years of disease, activity, and ongoing treatment.

### 3.5. Sources for Information and Clinicians’ Advice

**Figure 2:**
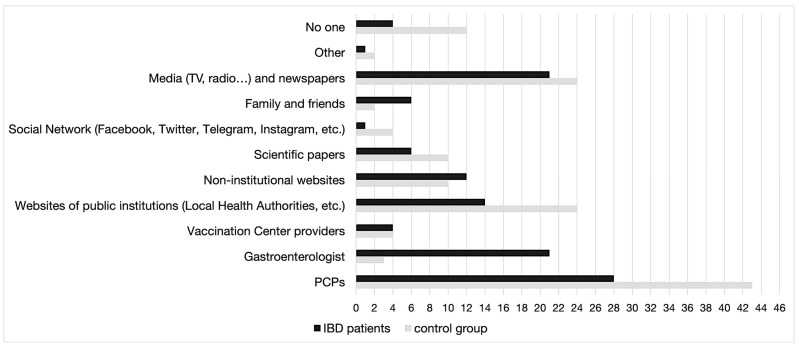
Main sources of information about COVID-19 immunization by number of preferences.

It was found that among the various sources of information available to IBD patients and controls about COVID-19 immunization (multiple preferences were allowed), PCPs were the most preferred source. However, gastroenterologists were more relevant for IBD patients. The media, newspapers, and websites of public institutions were also important sources of information. On the other hand, social networks were the least preferred option. Regarding the recommendations received from physicians to get vaccinated against COVID-19, it was found with statistical significance (*p* < 0.0001) that IBD patients relied heavily on their gastroenterologists (33.8%) for advice, while the control group relied mainly on their PCPs (44.9%). Moreover, a significant proportion of IBD patients and controls reported that they had not received any recommendation from medical professionals to get vaccinated against COVID-19 (IBD 33.7% vs. 53.8% control group) (Figure 2) (Table 5).

## 4. Discussion

Vaccine acceptance, defined as the individual or group decision to accept or refuse when presented with an opportunity to vaccinate [22], is a central topic in the area of public health. In 2019, the WHO Strategic Advisory Group of Experts (SAGE) listed the “delay in acceptance or refusal of vaccines despite availability of vaccination services” as one of the top ten global health threats. The issue is extremely relevant for IBD patients, who are at increased risk of becoming infected by several vaccine-preventable diseases [23].

The SARS-CoV-2 pandemic was characterized by vaccine diffidence induced by mass media misinformation and emphasis on the hypothetical side effects of vaccines [24].

Nonetheless, 79.2% of IBD patients in our study completed a two-dose series of the COVID-19 vaccine, which is higher than the 78.1% of the general population in Italy (as of December 2022) [25]. Our findings are consistent with the literature, which shows an 81% pooled prevalence of COVID-19 vaccine coverage in IBD patients worldwide for the entire vaccination regimen [26,27,28,29].

The proportion of non-IBD patients vaccinated with the complete series was significantly lower, attesting to 8%. Only 5.2% of IBD patients were not vaccinated, compared to 11.8% of the general Italian population [25]. It is worth noting that the number of participants who received their third dose at the time of the study was relatively small, as the national immunization schedule had only been recently implemented.

A limitation of the study consists of the higher proportion of non-IBD patients that refused to participate in the survey (2 IBD vs. 11 non-IBD patients), with a possible bias selecting controls that were more likely to get vaccinated.

In our cohort, no difference in the propensity to vaccinate was detected according to the diagnosis of CD or UC, disease duration, extent, and activity, as reported in previous studies [27].

No significant difference was found between cases and controls with regard to the overall attitude towards vaccines. Positive attitude towards vaccination in general was documented in 89.6% of our IBD participants. This is in line with data reported in a recent survey in Italian IBD patients [28] but differs from the high vaccine hesitancy rates reported in patients affected by other chronic/autoimmune diseases [30].

However, our data confirm low rates for seasonal influenza vaccination uptake among IBD patients (33.8%), comparable to those reported by previous studies (ranging from 28% to 61%) [31].

It can be speculated that the sub-optimal IBD vaccination rates against other infectious diseases before the COVID-19 pandemic resulted from physicians’ low attention to the issue and low patient awareness, more than patients’ negative attitudes. Indeed, several meta-analyses confirm that physicians’ interventions increase vaccination rates in IBD [32].

Health-care workers are a primary source of information, and physicians’ advice is crucial for patients’ vaccination compliance [33]. Several studies reported that inadequate attention to immunization among gastroenterologists [34,35,36] and primary care physicians (PCPs) [37] represents a major factor leading to low vaccination rates in IBD. Patients’ lack of awareness [23], fear of adverse secondary events [38], and doubts about the vaccines’ efficacy [39,40] have also been reported.

The COVID-19 vaccination campaign showed some peculiar characteristics only partly applicable to other diseases, reaching higher coverage and achieving heard immunity. This resulted from the fear of severe disease and unfavorable outcomes in the COVID-19 pandemic, as well as proactive intervention by attending physicians. Nonetheless, the overall positive attitude towards vaccinations reported in our study, as well as the relationship of trust with gastroenterologists, should boost efforts to optimize vaccination against other preventable diseases in IBD patients. Some vaccines are considered mandatory in IBD, including MMR (measles, mumps, and rubella) Varicella, Hepatitis B, pneumococcus, and influenza [41], and should be strongly encouraged in all IBD patients. The right timing is crucial in patients requiring treatment with immunomodulators and biologics as these therapies represent a contraindication to live vaccines and reduce the efficacy of inactivated vaccines.

Understanding attitudes and potential influencing factors is necessary to offer targeted vaccination-encouraging strategies and implement interventions to increase vaccination rates, especially considering the most recent possibility of an annual COVID-19 booster for long-term protection. This will benefit both patients and health-care providers as education is the only intervention that can effectively enhance vaccination rates [10].

## 5. Conclusions

Our research provides critical insight into the attitudes of IBD patients concerning vaccination, focusing on factors associated with propensity rather than hesitancy, as in most other studies.

Patients with IBD have an overall positive attitude towards vaccination, which was boosted by the SARS-CoV-2 pandemic. As vaccination rates were lower for other suggested vaccinations, such as influenza, great potential exists in IBD for optimizing immunization against other preventable diseases. As awareness of the issue and the trusting relationship with the treating gastroenterologist were central in favoring good adherence to the COVID-19 vaccination, the same approach should be applied in other settings and possibly in patients affected by other chronic diseases.

## Figures and Tables

**Table 1:** Sociodemographic characteristics of the included patients (N = 178).

	IBD Patients n = 77 (43.3%)	Control Group n = 101 (56.7%)	*p*-Value
**Sex, n (%)**			0.327
Male	37 (48.1)	56 (55.4)	
Female	40 (51.9)	45 (44.6)	
**Age (years), mean (±SD)**	49.5 (±15.4)	51.6 (±18.3)	
**Age group (years), n (%)**			0.128
18–39	19 (24.7)	29 (30.5)	
40–59	36 (46.8)	30 (31.6)	
>60	22 (28.5)	36 (37.9)	
**Highest level of educational attainment, n (%)**			0.973
Primary school	3 (3.9)	4 (4.0)	
Lower secondary school	15 (19.5)	18 (18.0)	
Upper secondary school	41 (53.3)	58 (58.0)	
Higher education	18 (23.3)	20 (20.0)	
**Occupation, n (%)**			0.050
Student	1 (1.3)	6 (6.2)	
Worker	46 (59.7)	46 (47.4)	
Retired	12 (15.6)	9 (9.3)	
Unemployed	18 (23.4)	36 (37.1)	
**Self-perceived health**			0.071
Poor	11 (14.3)	15 (14.9)	
Fair	41 (53.2)	37 (36.6)	
Good	19 (24.7)	43 (42.6)	
Very good	6 (7.8)	6 (5.9)	

**Table 2:** Clinical features of IBD patients (N = 77).

Diagnosis, n (%)		
Crohn’s disease	37 (48.1)	
Ulcerative colitis	40 (51.9)	
**Years of disease, mean (±SD)**	13.7 (±11.2)	
**Years of disease range, n (%)**		
<1	3 (3.9)	
1–5	26 (33.8)	
6–10	8 (10.4)	
11–15	12 (15.6)	
16–20	6 (7.8)	
>20	22 (28.5)	
**CD location, n (%)**		
Ileal	10 (27.0)	
Colonic	4 (10.8)	
Ileocolonic	23 (62.2)	
Isolated upper disease	0 (0.0)	
**UC extent, n (%)**		
Proctitis	1 (2.5)	
Left-sided	15 (37.5)	
Extensive disease	24 (60.0)	
**Activity, n (%)**	CD	UC
Quiescent	20 (54.1)	28 (70)
Mild	12 (32.4)	8 (20.0)
Moderate	5 (13.5)	3 (7.5)
Severe	0 (0.0)	1 (2.5)
**BSG risk grid, n (%)**		
Lowest risk	19 (24.7)	
Moderate risk	49 (63.6)	
Highest risk	9 (11.7)	
**Treatment, n (%)**		
Biologic therapy	34 (44.2)	
Conventional therapy	43 (55.8)	

**Table 3:** Personal history of COVID-19 and assessment of vaccine uptake (N = 178).

	IBD Patients n = 77 (43.3%)	Control Group n = 101 (56.7%)	*p*-Value
**Tested positive for COVID-19, n (%)**			0.0464
yes, asymptomatic infection	3 (3.9)	2 (2.0)	
yes, mild symptoms	4 (5.2)	22 (22.0)	
yes, moderate symptoms	1 (1.3)	3 (3.0)	
yes, severe symptoms/hospitalization	1 (1.3)	2 (2.0)	
no	68 (88.3)	71 (71.0)	
**Personally knew anyone with COVID-19, n (%)**			0.0886
Yes	48 (62.3)	76 (77.6)	
No	29 (37.7)	23 (22.4)	
**Received COVID-19 vaccine, n (%)**			<0.0001
yes, 1 dose	8 (10.4)	92 (91.0)	
yes, 2 doses	61 (79.2)	9 (9.0)	
yes, 3 doses	4 (5.2)	0 (0.0)	
not been vaccinated	4 (5.2)	0 (0.0)	
**Vaccinated for influenza, n (%)**	25 (33.8)	49 (66.2)	0.082

**Table 4:** Estimates of variables correlated to COVID-19 vaccine propensity.

	IBD Patients n = 77 (43.3%)		Control Group n = 101 (56.7%)		Differences between Correlations
	Correlation Estimate (95% CI)	*p*-Value	Correlation Estimate (95% CI)	*p*-Value	*p*-Value
I am vaccinated against COVID-19	0.16 (−0.06 to 0.38)	0.1603	−0.27 (−0.27 to −0.08)	0.0057	0.002
Vaccines can prevent severe illness	0.60 (0.43 to 0.73)	<0.0001	0.43 (0.25 to 0.58)	<0.0001	0.07
COVID-19 vaccination will make the pandemic end sooner	0.73 (0.60 to 0.83)	<0.0001	0.64 (0.50 to 0.75)	<0.0001	0.135
I believe in COVID-19 vaccine effectiveness	0.77 (0.65 to 0.85)	<0.0001	0.69 (0.57 to 0.78)	<0.0001	0.153
I’m concerned about the safety of COVID-19 vaccination in IBD	−0.28 (−0.48 to −0.04)	0.0202			
COVID-19 vaccination may trigger IBD flares	−0.28 (−0.48 to −0.04)	0.0192			
Type of IBD	0.07 (−0.16 to−0.30)	0.5479			
CD location	0.28 (−0.05 to 0.56)	0.0962			
UC extension	−0.15 (−0.46 to 0.19)	0.3902			
Years of disease	0.12 (−0.11 to 0.35)	0.2967			
IBD activity	−0.13 (−0.36 to 0.09)	0.2475			
Treatment	0.08 (−0.15 to 0.31)	0.4985			

**Table 5:** Physicians’ advice to get COVID-19 vaccine, n (%) (N = 178).

	IBD Patients n = 77 (43.3%)	Control Group n = 101 (56.7%)	*p*-Value
**Physicians’ advice to get COVID-19 vaccine, n (%)**			<0.0001
PCPs	18 (23.4)	35 (44.9)	
Gastroenterologists	26 (33.8)	1 (1.3)	
Other medical field professional/different specialization	4 (5.1)	0 (0.0)	
No one	23 (33.7)	42 (53.8)	

## Data Availability

The original contributions presented in the study are included in the article, further inquiries can be directed to the corresponding author.
